# A new anchialine *Stephos* Scott from the Yucatan Peninsula with notes on the biogeography and diversity of the genus (Copepoda, Calanoida, Stephidae)

**DOI:** 10.3897/zookeys.671.12052

**Published:** 2017-04-26

**Authors:** Eduardo Suárez-Morales, Martha A. Gutiérrez-Aguirre, Adrián Cervantes-Martínez, Thomas M. Iliffe

**Affiliations:** 1 El Colegio de la Frontera Sur (ECOSUR)-Chetumal, A.P. 424. Chetumal, Quintana Roo 77014, Mexico; 2 Universidad de Quintana Roo Campus Cozumel. Cozumel, Quintana Roo Mexico; 3 Department of Marine Biology. Texas A&M University at Galveston, Galveston, TX 77553-1675, USA

**Keywords:** Calanoid copepods, stygobionts, cave-dwelling fauna, biogeography, taxonomy

## Abstract

Surveys of the anchialine crustacean fauna of the Yucatan Peninsula (YP), Mexico, have revealed the occurrence of calanoid copepods. The genus *Stephos* Scott, 1892, belonging to the family Stephidae is among the most frequent and widely distributed groups in anchialine caves but has not been hitherto recorded from the YP. Recent collections from an anchialine cave in an island off the northern coast of the YP yielded many specimens of a new species of *Stephos*. The new taxon, *S. fernandoi* **sp. n.**, is described here based on male and female specimens. The new species is clearly distinguished from its congeners by the following characters: male left fifth leg with three terminal lamellae plus subdistal process, right leg with distal row of peg-like elements; female fifth leg with single long, acute apical process; genital double-somite with two rows each of 4 long spinules adjacent to operculum; legs 2-4 with articulated setae. The diversity of the genus shows regional differences; the Australia-Western Pacific region is the most diverse (eleven species), followed by the Mediterranean (seven species) and the Northeastern Atlantic (six species); only four species are known from the Northwestern Tropical Atlantic (NWTA). The morphology of the female fifth leg was examined to explore possible biogeographic trends in the genus; patterns suggest multiple colonization events in the highly diverse regions and a relatively recent radiation in the NWTA, characterized by anchialine forms. The introduction of stephid copepods in the region may be a relatively recent event derived from colonization of benthopelagic ancestral forms and subsequent invasion onto cave habitats. The new species appears to be linked to the strictly anchialine *Miostephos*.

## Introduction

The primitive calanoid copepod families Epacteriscidae and Ridgewayiidae are the most representative and diverse copepods in anchialine and subterranean environments worldwide (Fosshagen et al. 2001). Other calanoid families with cave-dwelling species are the Arietellidae, Pseudocyclopiidae, Pseudocyclopidae, Fosshageniidae, and Stephidae (Boxshall et al. 1990; Suárez-Morales and Iliffe 1996; Jaume et al. 1999; Fosshagen and Iliffe 2004). The latter family contains marine hyperbenthic forms living in coastal waters and in anchialine habitats. It contains 4 valid genera of which *Stephos* T. Scott, 1892 is the most diverse, currently incorporating 31 species (Bradford-Grieve 1999; Boxshall and Halsey 2004; Moon et al. 2015; Boxshall and Walter 2016). Members of this genus have been reported from tropical to polar latitudes worldwide; *Stephos* has been recognized to frequently inhabit submarine and anchialine caves (Jaume et al. 2008; Kršinić 2012, 2015). The remaining three stephid genera are relatively small, with a restricted distribution; together they contain a total of 6 species (Razouls et al. 2015, 2016). Previous reports of the family Stephidae from the Northwestern Tropical Atlantic (NWTA) include only a few species: *Stephos deichmannae* Fleminger, 1957 from surface plankton in the Gulf of Mexico (Fleminger 1957; Suárez-Morales et al. 2009), *S. lucayensis* Fosshagen, 1970, and *S. exumensis* Fosshagen, 1970, both from bottom samples of the Bahamas (Fosshagen 1970). The two known species of the genus *Miostephos* Bowman, 1976, *M. cubrobex* Bowman, 1976 from Cuba and *M. leamingtonensis* Yeatman, 1980 from Bermuda are other anchialine stephids from the NWTA (Razouls et al. 2015, 2016).

The anchialine crustacean fauna of the Yucatan Peninsula (YP) of Mexico is widely recognized as highly interesting, with many endemic species (Yager 1987; Iliffe 1992; Mercado-Salas et al. 2013; Boxshall et al. 2014). Members of the Ridgewayiidae (Suárez-Morales and Iliffe 2005) and Epacteriscidae (Suárez-Morales et al. 2006) have been hitherto recorded in the YP, but there are no data on the occurrence of other anchialine calanoid families. During a biological survey of the crustacean fauna of an anchialine cave in the island of Cozumel, off the northeastern coast of the YP, many male and female specimens of copepods were collected. A first analysis revealed the presence of a calanoid tentatively identified as belonging to the family Stephidae. A detailed examination revealed that these specimens represent an undescribed species of the genus *Stephos* which is herein described in full and compared with its known congeners. The distribution and diversity of the genus in the NWTA is also analyzed.

## Materials and methods

Specimens were collected on 6 July 2014 during a biological survey of an anchialine cave, Cenote Tres Potrillos, on Cozumel Island at 20°27'3.2"N, 86°59'14.4"W, Quintana Roo, Mexico. From a small pool at the cave entrance, a vertical shaft opens into a very large chamber with a halocline at 11 m. Beneath the halocline, sulfidic, fully marine water reaches a maximum depth of 38 m. A 40 m long passage at 12 m depth extends off the side of the main chamber (Mejía et al. 2008, fig. 3). Other anchialine crustaceans from this cave includes the shrimp *Barbouria yanezi* Mejía, Zarza & López, 2008, *Agostocaris* sp., and *Procaris* sp., the isopod *Bahalana* sp., and the amphipod *Mayaweckelia* sp. Plankton specimens were collected in the halocline with the aid of a conical plankton net (50 µm mesh size). The collected material was fixed and preserved in 100% ethanol. The copepods were sorted from the original sample and then transferred to glycerol. Specimens were prepared for taxonomic analysis by dissecting all appendages and light staining them with Methylene blue; the appendages were examined as temporary mounts in glycerine and sealed with Entellan® as permanent mounts. Drawings were prepared using a camera lucida mounted on an E-200 Nikon compound microscope with Nomarski DIC at magnifications of 1000X. Male and female specimens were prepared for SEM examination with a JEOL SM-6010 microscope at facilities of ECOSUR in Chetumal, Mexico. The process included dehydration of specimens in progressively higher ethanol solutions (60, 70, 80, 96, 100%) and drying with a treatment with hexamethyldisilazane (HMDS). Terminology of the body parts and appendages followed Huys and Boxshall (1991). Body length was measured from the anterior margin of the cephalosome to the posterior margin of the caudal rami. The type specimens are deposited in the collection of zooplankton held at El Colegio de la Frontera Sur (ECOSUR), Chetumal, Quintana Roo, Mexico (ECO-CHZ) and in the National Museum of Natural History, Smithsonian Institution (USNM) (MSC, Maryland), United States. Original samples are deposited at the Universidad de Quintana Roo (UQROO) Campus Cozumel, Mexico.

## Results

### Order Calanoida Sars, 1903

#### Family Stephidae T. Scott, 1892

##### Genus *Stephos* T. Scott, 1892

###### Stephos fernandoi sp. n.

Taxon classificationAnimaliaCalanoidaStephidae

http://zoobank.org/DD961DAC-CECF-49A2-86D7-30A9E13270A1

**Figure 1.**
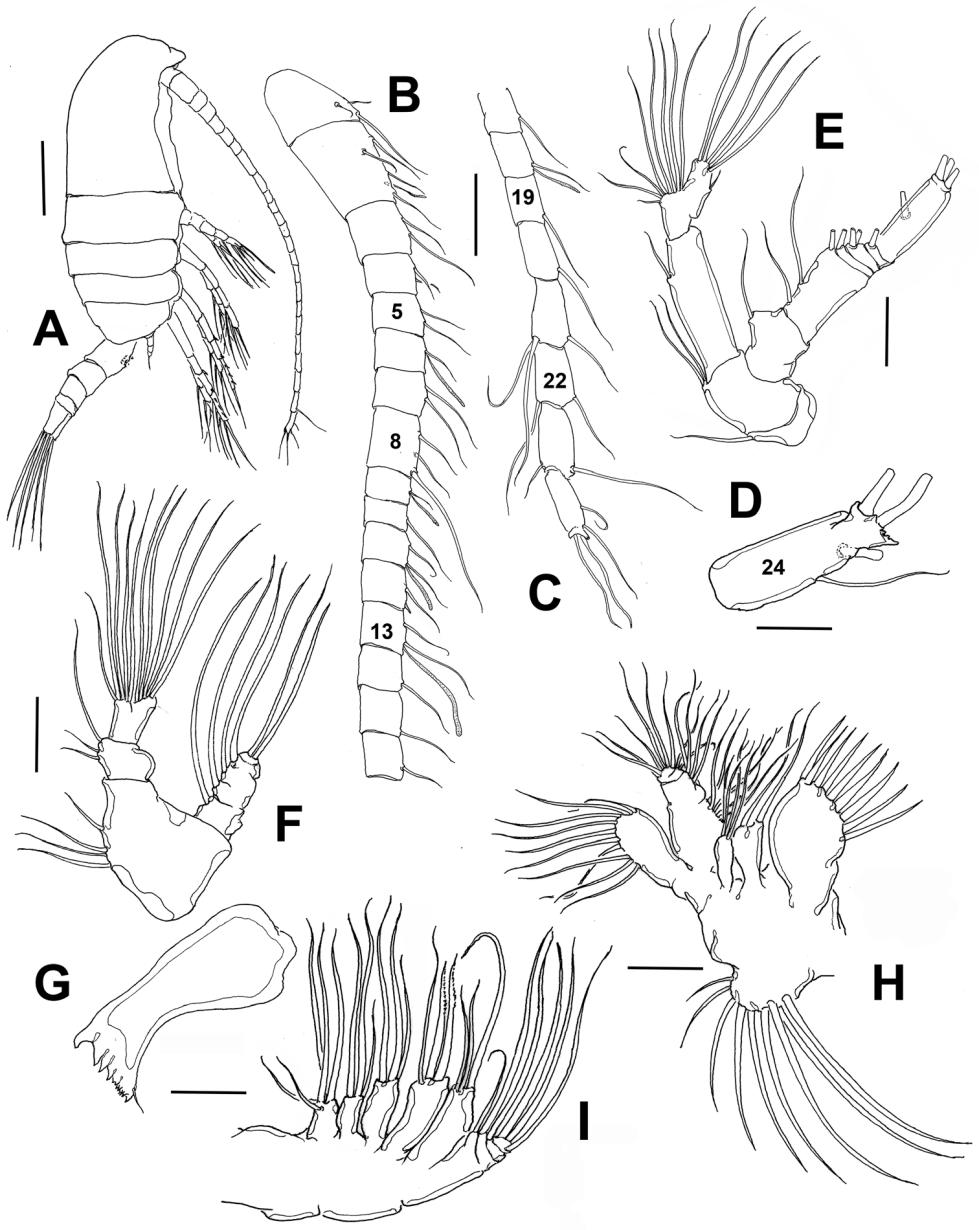
*Stephos fernandoi* sp. n., adult female from Cozumel, Mexico. **A** habitus, lateral view **B** antennule, segments 1–16 **C** antennule segments 17–24 **D** antennule segment 24 showing apical process **E** antenna **F** mandibular palp **G** gnathal base **H** maxillule **I** maxilla. Scale bars: **A** 100 μm; **D** 10 μm; **B, C, E–I** 20 μm.

**Figure 2.**
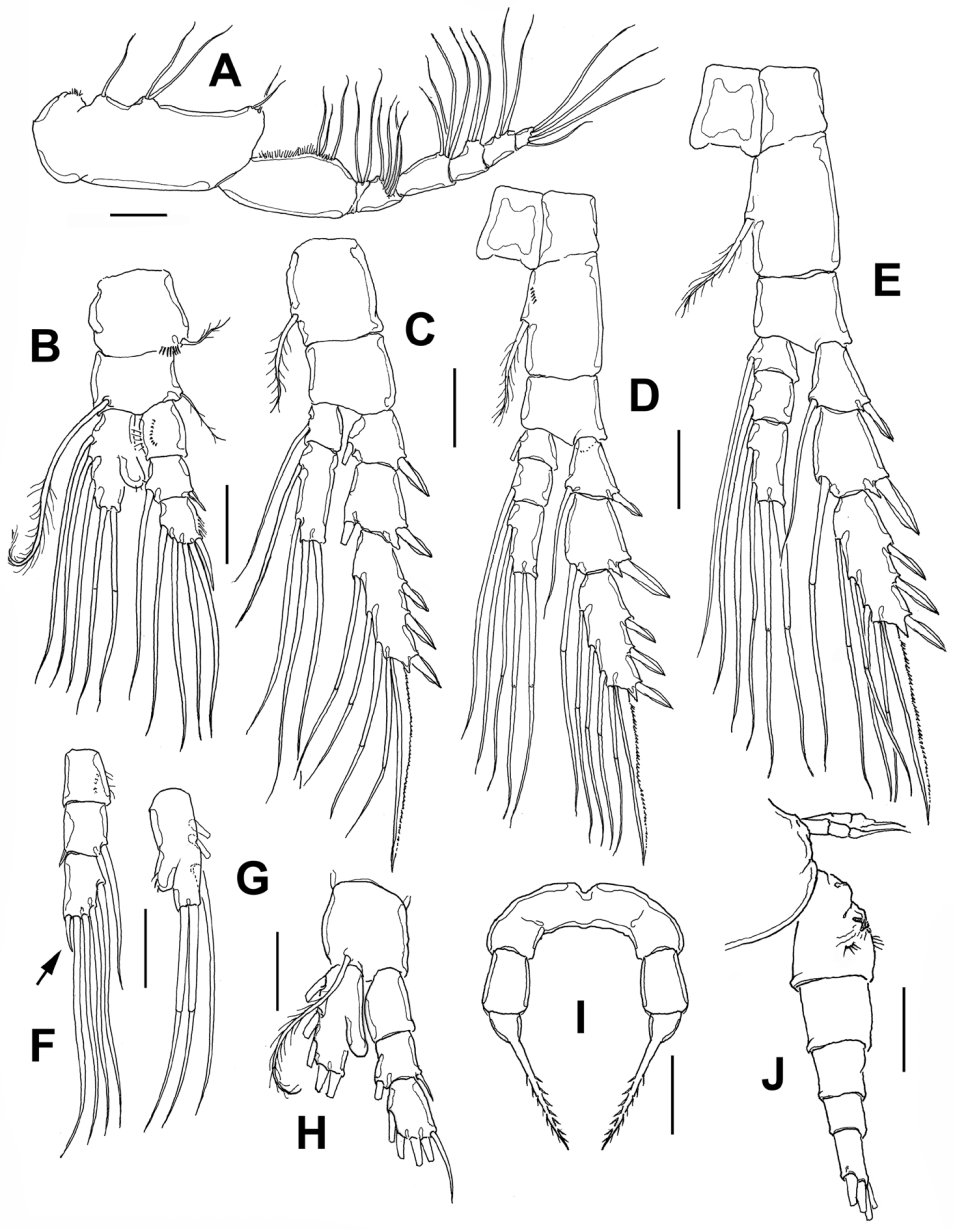
*Stephos fernandoi* sp. n., adult female from Cozumel, Mexico. **A** maxilliped **B** leg 1 **C** leg 2 **D** leg 3 **E** leg 4 **F** leg 1 exopod, another specimen showing reduced outermost apical spine (arrow) **G** leg 1 endopod, another specimen **H** male leg 1 **I** female leg 5, anterior view **J** fifth pedigerous somite and urosome, lateral view. Scale bars: **A–I** 20 μm; **J** 50 μm.

**Figure 3.**
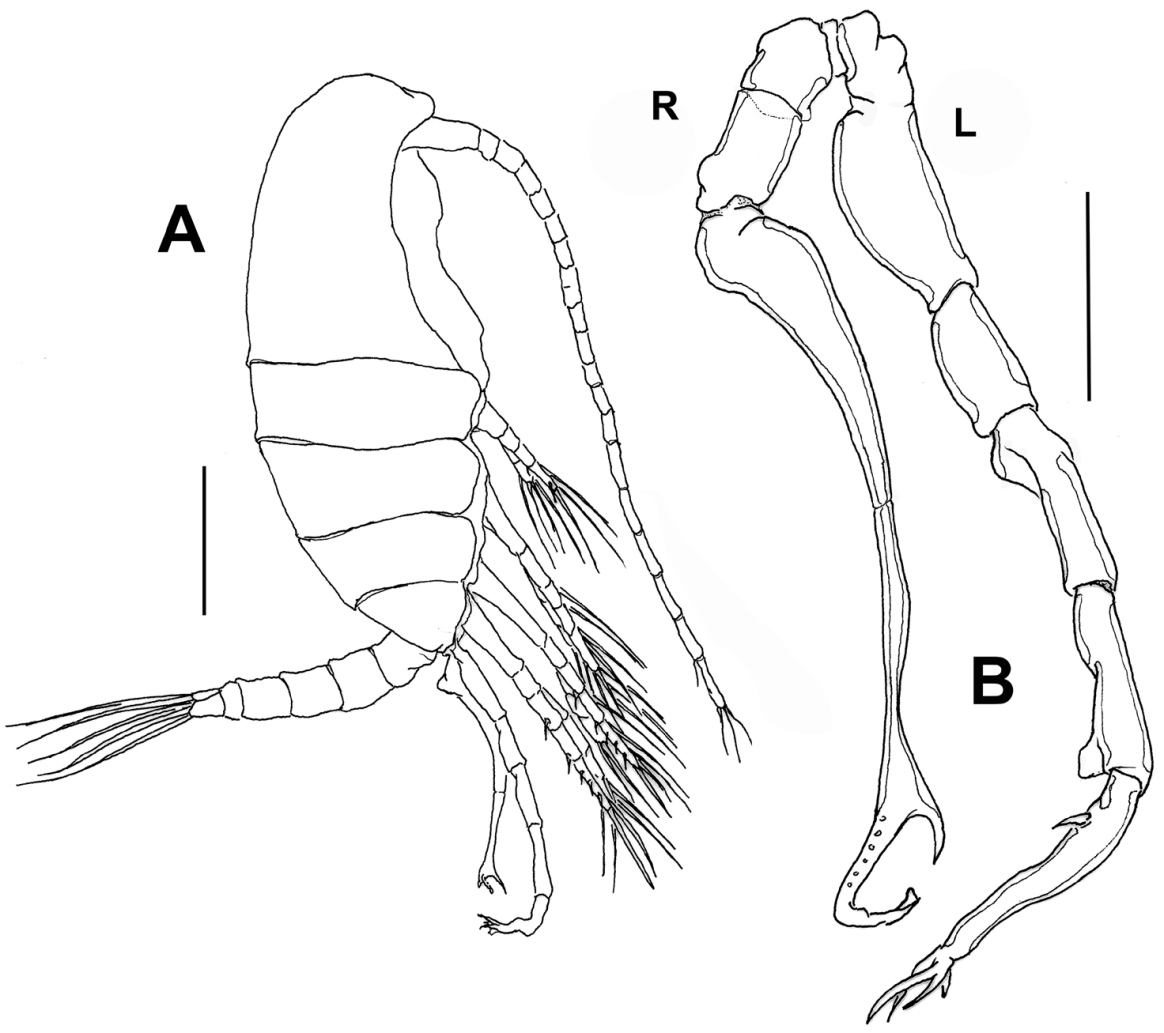
*Stephos fernandoi* sp. n., adult male from Cozumel, Mexico. **A** habitus, lateral view **B** leg 5. L=left ramus, R= right ramus. Scale bars: **A** 100 μm; **B** 50 μm.

**Figure 4.**
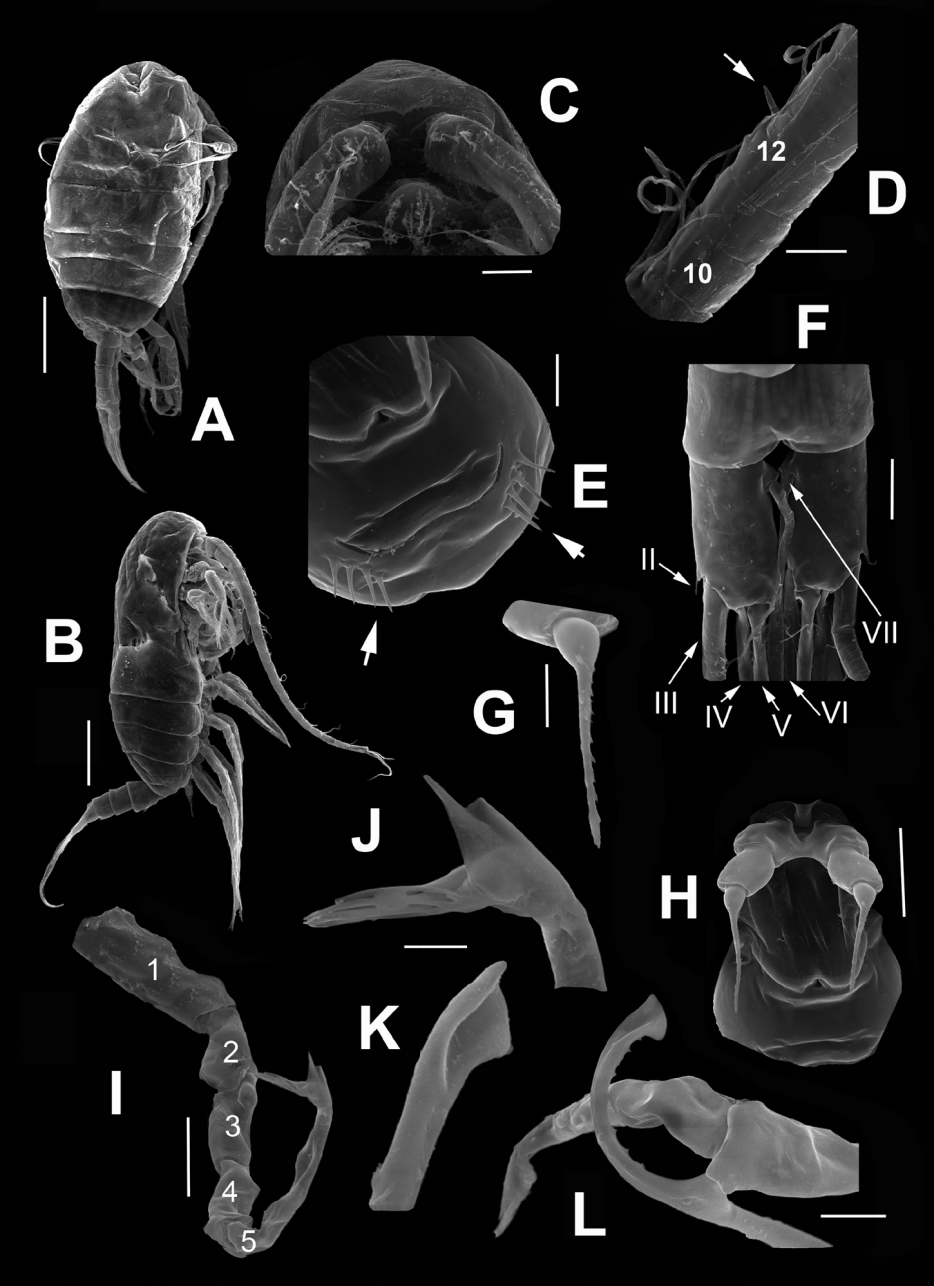
*Stephos fernandoi* sp. n., from Cozumel, Mexico. SEM-processed specimens. Female. **A** habitus, semi-lateral view **B** same, lateral view, another specimen **C** rostrum, ventral view **D** antennule segments 9–13, spiniform seta on segment 12 arrowed **E** genital double-somite, ventral surface showing ornamentation (arrows) **F** caudal rami showing caudal setae II-VII, ventral view **G** distal segment of leg 5 **H** leg 5, ventral view. Male. **I** left leg 5 showing segmentation **J** same, detail of distal segment **K** right leg 5, distal segment, detail of apical end **L** right leg 5, distal segment. Scale bars **A, B** 100 μm; **C, D, H, I** 20 μm, **E–G, L** 10 μm; **J, K** 5 μm.

Figures 1 , 2 , 3 , 4

####### Material examined.

Holotype. One adult ♀, collected on 6 July 2014 from the anchialine cave of Cenote Tres Potrillos, Cozumel Island (20°27'3.2"N, 86°59'14.4"W), Quintana Roo, Mexico. Specimen dissected on slide deposited in the collection of Zooplankton of El Colegio de la Frontera Sur (ECOSUR) in Chetumal, Mexico, under number ECO-CHZ-09411. Allotype: one adult ♂, collected on same date and site, specimen dissected (ECO-CHZ-09412). Paratypes: four dissected adult ♀♀, one dissected adult ♂, slides (ECO-CHZ-09413), two undissected ♀♀, eight undissected ♂♂ (ECO-CHZ-09414), and three undissected ♀♀, three ♂♂ (USNM-1422288), all from same date and site, ethanol-preserved, vials.

####### Descriptions.

*Female*. Mean length of prosome: 0.343 mm (*n* = 13); total length including caudal rami = 0.475 mm (*n* = 13). Body with typical calanoid shape, relatively robust in lateral and dorsal views, prosome 5-segmented, widest at first pedigerous somite (Figs 1A, 4A, B). Cephalosome and first pedigerous somite completely separate, fourth and fifth pedigerous somites fused, with posterolateral corners rounded, moderately produced, symmetrical (Fig. 4A). Rostrum weakly developed, represented by small medial expansion, rostral points absent (Fig. 4C). Urosome 4-segmented, representing 31% of total body length. Genital double-somite relatively long, almost 40% of urosome, barrel-shaped, symmetrical, weakly expanded mid-ventrally, expansion associated with genital field (Fig. 2J). Single gonopore opening ventrally at proximal 1/3 of somite; adjacent ventral surface of somite ornamented with 4 slender spiniform elements (arrowed in Fig. 4E) inserted at each side of simple, transverse genital operculum. Anal somite shortest of urosome, subrectangular, about 10% of urosome length, cuticular ornamentations absent on dorsal and ventral surfaces (Figs 2J, 4F).

Caudal rami subrectangular, symmetrical, length/width ratio = 1.6–1.7, armed with 6 caudal setae (II-VII) (Fig. 4F). Inner margin naked except for displaced dorsal seta (VII) inserted on proximal 1/4 of inner margin, seta reaching beyond distal margin of ramus (Fig. 4F). Caudal seta I absent, seta II (Fig. 4F) reduced, inserted near base of seta III. Terminal setae III-VI well developed. All ramal setae biserially plumose.

*Antennule* (Fig. 1B–D) 24-segmented, reaching posterior margin of preanal somite. Armature per segments as follows: segmental number (ancestral segment, setae (s) + aesthetasc (ae)): 1(I-II, 3s); 2(III-IV,4s + ae), 3(V, 2s), 4(VI, 2s), 5(VII, 2s), 6(VIII, 1s + ae), 7(IX, 2s), 8(X-XI, 3s), 9 (XII, 1s+ae), 10 (XIII, 1s), 11(XIV, 2s + ae), 12(XV, 2s), 13(XVI, 2s + ae), 14(XVII, 1s), 15(XVIII, 1s), 16(XIX, 1s), 17(XX, 1s), 18(XXI,1s + ae), 19 (XXII,1s), 20(XXIII,1s), 21(XXIV,2s +1s), 22(XXV,1s +1s), 23(XXVI, 1s +1s), 24(XXVII-XXVIII, 3s + ae) (Figs 1B–D). One of the setal elements on segment 12 spiniform (arrow in Fig. 4D). Distal segment with apical acute process present in some specimens (Fig. 1D).

*Antenna* (Fig. 1E) biramous, with exopod longer than endopod. Coxa armed with one seta. Basis with two distal subequal setae on medial margin. Endopod 2-segmented, first segment long, cylindrical, with short seta inserted at 2/3 of medial margin; distal portion of terminal segment with two lobes, proximal lobe with 8 setae; distal lobe with single short, lateral seta plus five long terminal setae. Exopod indistinctly 7-segmented, first segment with one long seta, second segment longest, armed with three setae, one proximal, one medial and one on distal position. Segments 3–6 with 1, 2, 1, 1 setae, respectively. Distal segment with crown of three long, terminal setae, subequal in length and diameter.

*Mandible* (Fig. 1G) with gnathobase armed with four large monocuspid ventral teeth plus three smaller bicuspid teeth, dorsal monocuspid tooth, and short dorsal seta. Serial teeth distinctly separated from large ventralmost tooth by diastema. Palp biramous (Fig. 1F), basis robust, armed with four subequal setae inserted on medial margin. Endopod short, 2-segmented; proximal segment with two short and one long setae, outer margin protuberant; distal segment subrectangular, with 10 setae, one reduced. Exopod indistinctly 5-segmented, armed with 1, 1, 1, 1, 2 setae.

*Maxillule* (Fig. 1H) with praecoxal arthrite bearing nine spiniform marginal setae. Coxal epipodite with nine setae, coxal endite with two setae. Basis with proximal endite bearing four setae, distal basal endite armed with five setae. Endopod reduced, not articulated to basis, indistinctly 3-segmented, proximal segment with four setae, second segment with two setae, distal segment with six. Exopod oblong, with ten subequal setae.

*Maxilla* (Fig. 1I) indistinctly 6-segmented including precoxa, coxa, allobasis and 3-segmented endopod. Praecoxal and coxal endites with 5, 3, 3, 3 setae, distal coxal endite with two stout spinulated setae. Basal endite of allobasis with 3 setae, incorporated endopodal segment with single seta. Free endopodal segments armed with 1, 1, 3 setae.

*Maxilliped* (Fig. 2A) indistinctly nine-segmented, precoxa and coxa partially fused, precoxa unarmed, with cluster of spinules. Coxa with three groups of setae, proximal endite with 1 seta, middle endite with two, distal with two. Basis ornamented with row of short spinules; armed with 3 setae, one shorter than the rest. Endopod six-segmented, armed as follows: 2, 4, 4, 2, 2, 4. Basal and endopodal setae slender, distally attenuated.

*Legs 1-4* (Fig. 2B–E) biramous, increasing in size posteriorly. First swimming leg (Fig. 2B) with three-segmented exopod and one-segmented endopod; coxa subrectangular, with short outer coxal seta not reaching distal margin of basal segment; row of spinules at insertion of coxal seta. Basipod with long, recurved inner plumose seta reaching beyond distal margin of third exopodal segment; outer basipodal seta slender. Endopod with outer knob ornamented with 1–3 minute apical setules (Fig. 2B, G). First exopodal segment with row of spinules. Outer spine on third exopodal segment elongate, spine shorter in some specimens (arrowed in Fig. 2F). Second leg with two-segmented endopod (Fig. 2C), legs 3 and 4 with three-segmented exopods and endopods, with articulate setae (Fig. 2D, E). Armature formula of legs 1–4 as in Table 1.

*Fifth legs* (Fig. 2I) reduced, symmetrical, uniramous, two-segmented with proximal segment cylindrical, distal segment proximally globose, forming long spiniform bipinnate apical process (Figs 2I, 4G, H).

*Male*. Body slightly longer than female, average total length: 0.493 mm (*n* =10); length of prosome: 0.31 mm (Fig. 3A). Rostrum as in female. Urosome 5-segmented, representing 32% of total body length. First urosomite symmetrical; anal somite shortest. Caudal rami relatively short, symmetrical, caudal setae as in female.

Left and right antennules 24-segmented, lacking geniculation, slightly longer than in female when extended posteriorly; antennulary armature as in female. Mouthparts and swimming legs 1–4 as in female.

Fifth legs (Figs 3B, 4I–L) uniramous, asymmetrical. Left leg five-segmented, about as long as right counterpart; proximal segment widest of ramus, with inner margin expanded. Second, third, and fourth segments elongate, fourth with triangular plate on distomedial angle; distal segment with three terminal lamellae tapering distally plus subdistal subtriangular process, and with short seta inserted proximally on medial margin (Fig. 4J). Right fifth leg (Fig. 3B) four-segmented, first and second segments cylindrical, robust, unarmed. Third segment elongate and tapering. Fourth segment very slender and bifurcating distally into “C”-shaped structure furnished with 6–8 peg-like elements along bifurcation (Fig. 4L). End of subdistal process acute, opposite end with apical leaf-like expansion (Fig. 4K).

####### Etymology.

The new species is named after the Mexican carcinologist Dr. Fernando Alvarez (Instituto de Biología, UNAM, Mexico), who has significantly contributed to the knowledge of the Mexican crustacean fauna, particularly from caves and anchialine habitats.

####### Remarks.

The new species was included in the diverse stephid genus *Stephos* based on its possession of the following characters: 1) cephalosome and first pedigerous somite separate, pedigers 4–5 partially fused, 2) female urosome 4-segmented, male five-segmented; 3) caudal rami with 4 terminal setae (III–VI), dorsal caudal seta VII inserted on inner margin; 4) antennules 24-segmented in male and female, lacking geniculation in male; 5) female leg 5 uniramous, one or two-segmented, distal segment tapering, ornamented; 6) male fifth legs uniramous, strongly asymmetrical, modified into grasping organ, left leg five-segmented, with complex distal segment, right leg slender (Bradford-Grieve 1999).

Based on the morphology of the male fifth legs, Bradford-Grieve (1999) divided the species of *Stephos* into four distinct groups. The new species *S. fernandoi* can be assigned to “group IV” by its possession of a male right leg 5 with a narrow fourth segment. Currently, this group includes nine species: *S. pentacanthos* Chen & Zhang, 1965 from off China, *S. tsuyazakiensis* Tanaka, 1966 from Japan, *S. rustadi* Strömgren, 1969 from Norway, *S. morii* Greenwood, 1977 from Australia, *S. pacificus* Ohtsuka & Hiromi, 1987 from Japan, *S. angulatus* Bradford-Grieve, 1999 from New Zealand, *S. marsalensis* Costanzo, Campolmi & Zagami, 2000 from Italy, *S. vivesi* Jaume, Boxshall & Gràcia, 2008 from the Balearic Islands, and *S. goejinensis* Moon, Yeon & Venmathi Maran, 2015 from Korea (see table 1 in Bradford-Grieve 1999; Jaume et al. 2008; Moon et al. 2015).

**Table 1.** Armature formula of swimming legs 1-4. Roman numerals indicate spines and Arabic numerals are setae.

	coxa	basis	exopod	endopod
leg 1	0-1	1-1	0-0; I-1; I,2,2	0,2,3
leg 2	0-1	0-0	I-1; I-1; III,I,4	0-1; 0,2,2
leg 3	0-1	0-0	I-1; I-1; III,I,4	0-1; 0-1; 0,2,2
leg 4	0-1	0-0	I-1; I-1; III,I,4	0-1; 0-1; 0,2,2

The new species is the only one in this group with a right leg 5 ramus combining a distal segment (segment 4) with diverging processes set at right angles with acute tips plus a series of peg-like elements along the longest process (Fig. 4L). It differs from *S. angulatus* because in this species, the processes are both apically rounded and the segment lacks the peg-like elements observed in the new species. The left ramus has a similar structure in both species, with segment 4 bearing a distal lobular process (Bradford-Grieve 1999, fig. 8; Fig. 4L) and three distal lamellae, but the new species has an additional subdistal process (Figs 3B, 4J). In *S. marsalensis*, the distal segment of right male P5 is unbranched (Costanzo et al. 2000, fig. 4d), thus diverging from the bifid condition found in *S. fernandoi*; also, the left leg has five lamellate hyaline processes on the distal segment vs. only three such processes in the new species. The anchialine *S. vivesi* has a left leg with eight narrow lamellae and a relatively simple, spatulate distal segment of the right leg with two proximal processes (Jaume et al. 2008, fig. 2b–d), thus diverging from the pattern observed in the new species. The fifth leg of the new species differs from that of *S. geojinensis* in the number of lamellae on the distal segment of the left leg, three (Fig. 4J) vs. seven long plus 13 short lamellae, and right leg with distal segment bifurcate, with both branches subequally long (Fig. 3B) vs. outer branch extremely long, inner branch reduced (Moon et al. 2015, fig. 4d). The same kind of distally asymmetrical right fifth leg is present in *S. tsuyazakiensis* (Tanaka 1966, fig. 1o), thus differing from the new species. In *S. morii*, the right leg distal segment has a strong inner bulb-like process (Greenwood 1977, fig. 4g) which is absent in the new species; also, the left leg terminal segment has a long, distinctive spiniform process on subdistal position which is not present in *S. fernandoi* (Fig. 3B). In *S. rustadi*, the right leg distal segment is chela-like, with an expanded inner margin and the left leg is clearly shorter that its right counterpart and has three distinctive hook-like processes (Strömgren 1969, fig. 3f), thus diverging from the fifth leg structure of the new species. In *S. penthacanthos*, the right fifth leg has a spiniform process on the outer margin and the terminal segment is modified into a long, slender claw-like process, curved inwardly (Chen and Zhang 1965, fig. 20.5), thus differing from the spatulate distal segment described in the new species. In *S. pacificus*, the right fifth leg distal segment is relatively simple, represented by an elongate, narrow unbranched structure (Ohtsuka and Hiromi 1987, fig. 3f), different from the pattern observed in the new species, clearly branched (Fig. 3B).

The female fifth leg of *S. fernandoi* differs from that known in most species of *Stephos*, which has a medial seta and/or a row of spinules on the distal segment. It most closely resembles the fifth legs of the two species of *Miostephos*, *M. cubrobex* from Cuba (Bowman 1976, fig. 13) and *M. leamingtonensis* from Bermuda (Yeatman 1980, fig. 5), both with an attenuated unarmed distal segment. The new species differs in the genus characters (i.e. three-segmented female urosome, six-segmented left male fifth leg, reduced male right fifth leg strongly resembling the female fifth leg) (Bowman 1976). In species of *Stephos*, the female genital double-somite has widely different patterns of ornamentation on the ventral and/or lateral surfaces, including rows of spinules with both symmetrical and asymmetrical arrangements (Ohtsuka and Hiromi 1987; Bradford-Grieve 1999; Costanzo et al. 2000), lack of surface ornamentation, as in *S. canariensis* (Boxshall et al., 1990) or *S. grieveae* (Kršinić, 2015) or a highly modified, strongly asymmetrical somite as in *S. exumensis* (Fosshagen, 1970). The new species has a unique pattern combining a symmetrical genital double-somite with an ornamentation pattern represented by a set of 4 spiniform elements at each side of the genital operculum; this pattern has not been observed in any other species of *Stephos*.

**Figure 5.**
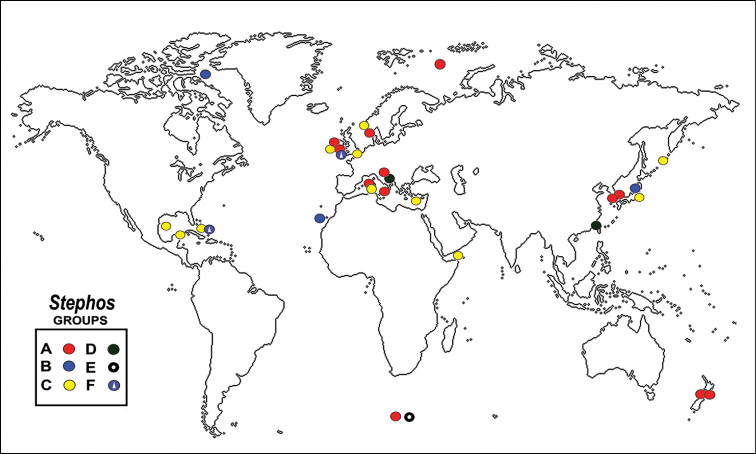
Geographic distribution of *Stephos* female fifth leg groups **A–F**. Group **A** lateral seta present, segment apically elongate **B** lateral seta present, segment not apically elongate, short **C** lateral seta absent, segment apically elongate **D** lateral seta present, segment elongate, with additional segmental processes, branched or bifurcate **E** lateral seta absent, segment elongate, with outer segmental processes **F** lateral seta absent, segment short, apically truncate or blunt, leg rami symmetrical or asymmetrical.

## Discussion

The distribution of primitive anchialine copepods (i.e., epacteriscids, misophrioids) in the Caribbean, Yucatan, the Canary Islands, and the tropical Pacific has been taken to indicate their Tethyan origin (Fosshagen et al. 2001; Boxshall and Jaume 2000; Boxshall et al. 2014). There are, however, groups whose invasion of cave habitats and subsequent isolation into anchialine waters is related to more recent events. During the Pleistocene, when the eastern coast of the YP emerged (Iturralde-Vinent and MacPhee 1999), some stygobionts which already were established in caves became isolated by the overlying freshwater lens. Cozumel Island is the most recently emerged land of the YP (Vázquez-Domínguez and Arita 2010) so the new species may represent a secondary invasion and probably colonized recently the youngest epicontinental anchialine systems of the YP.

In terms of its known geographic distribution, the 32 known species of *Stephos* occur in different regions each harboring its own, distinctive diversity, as follows:

a) Australia-Western Pacific (eleven species): *S. penthacanthos*, *S. morii*, *S. tropicus* Mori, 1942, *S. tsuyazakiensis*, *S. pacificus* Ohtsuka & Hiromi, 1987, *S. angulatus*, *S. robustus*, *S. kurilensis* Kos, 1972, *S. hastatus* Bradford-Grieve, 1999, *S. geojinensis*, *S. projectus* Moon, Youn & Venmathi Maran, 2015.

b) Mediterranean (seven species): *S. gyrans* (Giesbrecht, 1893), *S. margalefi* Riera, Vives & Gili, 1991, *S. vivesi*, *S. cryptospinosus* Zagami, Campolmi & Costanzo, 2000, *S. marsalensis*, *S. boettgerschnackae* Kršinić, 2012, *S. grieveae* Kršinić, 2015.

c) Northeastern Atlantic (six species): *S. canariensis* Boxshall, Stock & Sánchez, 1990, *S. rustadi*, *S. minor* Scott, 1892, *S. scotti* Sars, 1902, *S. fultoni* Scott T. & A., 1898, *S. lamellatus* Sars, 1902.

d) Northwestern Tropical Atlantic (four species): *S. deichmannae*, *S. exumensis*, *S. lucayensis*, *S. fernandoi*.

e) Polar (three species): *S. antarcticum* Wolfenden, 1908, *S. longipes* Giesbrecht, 1902, *S. arcticus* Sars, 1909.

f) Indo-Pacific (one species): *S. maculosus* Andronov, 1974.

The genus is most diverse in the Australia-Western Pacific region, followed by the Northeastern Atlantic and the Mediterranean. There are no records of *Stephos* from the Southwestern and Southeastern Atlantic and the Eastern Pacific (Fig. 5). Regional endemism is high; there are no confirmed records regarding the occurrence of any species of *Stephos* in more than one of these regions (Razouls et al. 2015, 2016). The restricted distribution of the anchialine stephid genera and species in the NWTA region (Fosshagen 1970; Bowman 1976; Yeatman 1980) and in other areas with a rich anchialine fauna (i.e., Mediterranean) suggest that the new species, *S. fernandoi*, is endemic to Cozumel Island. *Stephos fernandoi* is the third species of anchialine calanoid copepod recorded in the YP and represents the second record of the family Stephidae in Mexican waters (Suárez-Morales et al. 2009).

Stephids are in general benthopelagic or anchialine forms, strongly associated with the bottom communities, but some species are known from the plankton (Fleminger 1957; Kos 1972; Ohtsuka and Hiromi 1987; Costanzo et al. 2000; Zagami et al. 2000; Moon et al. 2015). Except for the planktonic *S. deichmannae*, all the stephids found in the NWTA are cave-dwelling anchialine forms (Fosshagen 1970; Bowman 1976; Yeatman 1980). The availability of cave habitats in the NWTA region has favored a highly endemic stephid fauna, likely younger and less speciose but comparable with that of the Mediterranean.

### Morphological analysis and biogeography

In *Stephos*, the male fifth legs show at least four different morphological patterns (Bradford-Grieve 1999) but little can be inferred from them in terms of biogeographic patterns. In order to explore possible distributional trends in the genus, we examined and grouped the structural patterns of the female fifth leg of 29 species of *Stephos*. These morphological types were based on the development and armature of the distal segment, as follows: A) lateral seta present, segment apically elongate (*S. boettgerschnackae*, *S. cryptospinosus*, *S. angulatus*, *S. rustadi*, *S. vivesi*, *S. geojinensis*, *S. hastatus*, *S. lamellatus*, *S. minor*, *S. longipes*, *S. projectus*); B) lateral seta present, segment not apically elongate, short (*S. arcticus*, *S. canariensis*, *S. tsuyazakiensis*), C) lateral seta absent, segment apically elongate (*S. exumensis*, *S. deichmannae*, *S. maculosus*, *S. scotti*, *S. fernandoi*, *S. kurilensis*, *S. pacificus*, *S. marsalensis*, *S. gyrans*), D) lateral seta present, segment elongate, with additional segmental processes, branched or bifurcate (*S. grieveae*, *S. penthacanthos*); E) lateral seta absent, segment elongate, with outer segmental processes (*S. antarcticum*); F) lateral seta absent, segment short, apically truncate or blunt, leg rami symmetrical or asymmetrical (*S. margalefi*, *S. lucayensis*, *S. fultoni*).

Pattern D was deemed as the most primitive group, followed by groups A and B, also with a lateral seta and an apically elongate or short segment, respectively. The derived patterns are those lacking a lateral seta (i.e., E, C, and F). The known distribution of records of these six groups is presented in Fig. 5. Group D is restricted to the Mediterranean and Japan; the primitive patterns A and B are the most widespread, distributed in the most diverse regions (i.e., the Mediterranean, northeastern Atlantic) and reaching polar and subpolar latitudes. The derived groups C and F occur in the most diverse regions but they are the only groups present in the Western Hemisphere, restricted to the NWTA region. The distribution of the species diversity and our interpretation of the female fifth leg types suggest the occurrence of different colonization and speciation events in these coastal demersal copepods resulting from geological changes (i.e., marine regressions and transgressions) in each region (see Fleminger 1986; Por 1986; Suárez-Morales 2003). The genus probably passed through different episodes of diversification mainly in regions such as the western Pacific and the Mediterranean, where both primitive and derived patterns of the female fifth legs co-occur (Fig. 5). The complex biogeographic history of the Mediterranean explains the high diversity and co-occurrence of species belonging to at least three of our groups, suggesting distinct origins, and remarkably characterized by their preference for anchialine and cave habitats (Por 1986; Jaume et al. 2008; Kršinić 2015). Contrastingly, the most recent radiation of *Stephos* appears to have taken place in the NWTA, represented by only four species with a derived female fifth leg; three of them are anchialine (*S. lucayensis*, *S. exumensis*, *S. fernandoi*) (Fosshagen 1970; present data) and one is planktonic (*S. deichmannae*) (Fleminger 1957). Furthermore, the new species, *S. fernandoi*, has clear affinities with the two species of *Miostephos*, mainly in the reduced female fifth leg (as in pattern C) (Bowman 1976; Yeatman 1980); it is thus suggested that *Stephos*-like benthopelagic ancestors invaded the region, colonized the caves and one branch subsequently diverged into the *Miostephos* lineage in the NWTA. Similar colonization processes, with diversity/radiation centers in the western Pacific and congeners distributed in the NWTA have been described for other demersal calanoids like *Tortanus* (Ohtsuka & Reid, 1998) and *Bestiolina* (Suárez-Morales & Almeyda-Artigas, 2016). It is speculated that ancestors of *Stephos* probably originated in the Australian-Western-Pacific region and successively colonized the Mediterranean and the northeastern Atlantic. They probably reached the NWTA either through the Isthmus of Panama during the Middle Miocene-Pliocene (Ohtsuka and Reid 1998) although there are no records of *Stephos* from the eastern Pacific coast, or by passive transportation of planktonic forms onto the NWTA. This analysis should be considered tentative as there are still many missing data; a complete morphological revision of incompletely described species, a confirmation of doubtful records, and a phylogenetic analysis including molecular data is expected to reveal more detailed patterns about this genus.

## Supplementary Material

XML Treatment for Stephos fernandoi
